# Supplementary material for: Analysis of the Effect of Processing Conditions on Physical Properties of Thermally Set Cellulose Hydrogels
Source: Materials (Basel). 2019 Apr 1;12(7):1066. doi: 10.3390/ma12071066 (PMC6479291; doi:10.3390/ma12071066)
Supplement: Supplementary file 1 [file materials-12-01066-s001.zip › sample-list.pdf]

Sample formulation					Sample testing				
**Gelation method**	**ECH concentration**	**Added undisolved cellulose**	**Set temperature in deg C**	**cross-linking**	**Negative templating**	**FITR**	**Cross-linking efficency**	**Microstruture anlysis**	**Compression testing**
water bath	0%	0%	40	-	x			x	x
			50	-		x	x	x	x
			60	-				x	x
			70	-				x	x
			80	-				x	x
	0%	50%	50	during gelation		x			x
			60	during gelation					x
			70	during gelation					x
	0%	100%	70	during gelation					x
	5%	0%	50	during gelation					x
			70	during gelation					x
	10%	0%	50	during gelation					x
			70	during gelation					x
	15%	0%	50	during gelation					x
			70	during gelation					x
	5%	50%	50	during gelation		x			x
			70	during gelation					x
	10%	50%	50	during gelation & post gelation		x	x	x	x
			70	during gelation					x
	15%	50%	50	during gelation		x			x
			70	during gelation					x
Hot press	0%	0%	70	-					x
	0%	10%	70	during gelation				x	x
	0%	50%	50	during gelation					x
			70	during gelation & post gelation			x	x	x
	0%	100%	70	during gelation & post gelation			x	x	x
	5%	0%	50	during gelation					x
			70	during gelation & post gelation			x	x	x
	5%	10%	70	during gelation				x	
	5%	50%	50	during gelation					x
			70	during gelation & post gelation			x	x	x
	5%	100%	70	during gelation				x	
	10%	0%	50	during gelation					x
			70	during gelation & post gelation			x	x	x
	10%	10%	70	during gelation				x	
	10%	50%	50	during gelation					x
			70	during gelation & post gelation			x	x	x
	10%	100%	70	during gelation				x	
	15%	0%	50	during gelation					x
			70	during gelation & post gelation			x	x	x
	15%	10%	70	during gelation				x	
	15%	50%	50	during gelation					x
			70	during gelation & post gelation			x	x	x
	15%	100%	70	during gelation				x	
Microwave 100 W	0%	0%	-	during gelation				x	x
	0%	50%	-	during gelation					x
Microwave 180 W	0%	0%	-	during gelation				x	x
